# Presenilins regulate synaptic plasticity and mitochondrial calcium homeostasis in the hippocampal mossy fiber pathway

**DOI:** 10.1186/s13024-017-0189-5

**Published:** 2017-06-15

**Authors:** Sang Hun Lee, David Lutz, Mohanad Mossalam, Vadim Y. Bolshakov, Michael Frotscher, Jie Shen

**Affiliations:** 1 Department of Neurology, Brigham & Women’s Hospital, Harvard Medical School, Boston, MA 02115 USA; 2 Institute for Structural Neurobiology, Center for Molecular Neurobiology Hamburg (ZMNH), University Medical Center Hamburg-Eppendorf, D-20246 Hamburg, Germany; 3 Department of Psychiatry, McLean Hospital, Harvard Medical School, Belmont, MA 02478 USA; 4 Program in Neuroscience, Harvard Medical School, Boston, MA 02115 USA

**Keywords:** Presenilin, Mossy fiber, Synaptic plasticity, Mitochondria, Calcium

## Abstract

**Background:**

Presenilins play a major role in the pathogenesis of Alzheimer’s disease, in which the hippocampus is particularly vulnerable. Previous studies of Presenilin function in the synapse, however, focused exclusively on the hippocampal Schaffer collateral (SC) pathway. Whether Presenilins play similar or distinct roles in other hippocampal synapses is unknown.

**Methods:**

To investigate the role of Presenilins at mossy fiber (MF) synapses we performed field and whole-cell electrophysiological recordings and Ca^2+^ imaging using acute hippocampal slices of postnatal forebrain-restricted *Presenilin* conditional double knockout (*PS* cDKO) and control mice at 2 months of age. We also performed quantitative electron microscopy (EM) analysis to determine whether mitochondrial content is affected at presynaptic MF boutons of *PS* cDKO mice. We further conducted behavioral analysis to assess spatial learning and memory of *PS* cDKO and control mice at 2 months in the Morris water maze.

**Results:**

We found that long-term potentiation and short-term plasticity, such as paired-pulse and frequency facilitation, are impaired at MF synapses of *PS* cDKO mice. Moreover, post-tetanic potentiation (PTP), another form of short-term plasticity, is also impaired at MF synapses of *PS* cDKO mice. Furthermore, blockade of mitochondrial Ca^2+^ efflux mimics and occludes the PTP deficits at MF synapses of *PS* cDKO mice, suggesting that mitochondrial Ca^2+^ homeostasis is impaired in the absence of PS. Quantitative EM analysis showed normal number and area of mitochondria at presynaptic MF boutons of *PS* cDKO mice, indicating unchanged mitochondrial content. Ca^2+^ imaging of dentate gyrus granule neurons further revealed that cytosolic Ca^2+^ increases induced by tetanic stimulation are reduced in *PS* cDKO granule neurons in acute hippocampal slices, and that inhibition of mitochondrial Ca^2+^ release during high frequency stimulation mimics and occludes the Ca^2+^ defects observed in *PS* cDKO neurons. Consistent with synaptic plasticity impairment observed at MF and SC synapses in acute *PS* cDKO hippocampal slices, *PS* cDKO mice exhibit profound spatial learning and memory deficits in the Morris water maze.

**Conclusions:**

Our findings demonstrate the importance of PS in the regulation of synaptic plasticity and mitochondrial Ca^2+^ homeostasis in the hippocampal MF pathway.

## Background

Alzheimer’s disease (AD) is the most common age-related neurodegenerative disorder characterized by progressive memory loss and cognitive decline. Mutations in the *Presenilin* genes account for ~90% of all causative mutations in familial AD, highlighting their importance in AD pathogenesis. Genetic studies using conditional gene targeting approaches revealed that Presenilins are essential for learning and memory, synaptic function and age-dependent neuronal survival [1–3].

Synaptic dysfunction is widely thought to be one of the earliest key pathogenic events in AD before frank neurodegeneration [1, 4–6], and the hippocampal network is particularly vulnerable in AD [7–10]. The hippocampus consists of three main fields, dentate gyrus (DG), areas CA3 and CA1, and each field displays unique anatomical, molecular, and connectivity patterns [11, 12]. The tri-synaptic circuit conducts synaptic transmission in the hippocampus, and consists of three major excitatory synaptic pathways: perforant path (PP) → DG, mossy fiber (MF) → CA3, and Schaffer collateral (SC) → CA1 [13]. All three hippocampal pathways have been associated with learning and memory [14–16], and disruption of the hippocampal network has been implicated in AD. For example, structural and functional MRI analysis of AD patients revealed disruption of the MF-CA3 pathway in patients with mild AD or mild cognitive impairment [17, 18].

We previously reported that inactivation of Presenilins results in impairment of neurotransmitter release, short- and long-term synaptic plasticity at hippocampal SC synapses [1, 2, 19]. However, it was unknown whether Presenilins play similar or distinct roles in the regulation of synaptic function at other hippocampal synapses. In the current study, we focus on the hippocampal MF pathway using the postnatal forebrain-restricted *Presenilin* conditional double knockout (*PS* cDKO) mice, in which Presenilins are inactivated in excitatory neurons of the hippocampus beginning at postnatal day 18 [1, 2, 19, 20]. We found that long-term potentiation (LTP), paired-pulse facilitation (PPF) and synaptic facilitation are impaired at MF synapses in *PS* cDKO mice. Moreover, post-tetanic potentiation (PTP), which lasts longer than facilitation and results from the slow efflux of tetanically accumulated mitochondrial Ca^2+^ [21, 22], is also reduced at MF synapses in *PS* cDKO mice. Pharmacological blockade of mitochondrial Ca^2+^ efflux mimics and occludes PTP deficits at MF synapses of *PS* cDKO mice, suggesting an impairment of mitochondrial Ca^2+^ at MF synapses in the absence of Presenilins. However, quantitative electron microscopy (EM) analysis showed similar numbers and areas of mitochondria between control and *PS* cDKO mice at hippocampal MF presynaptic terminals. Consistent with these findings, Ca^2+^ imaging of DG granule neurons showed that repeated stimulation-induced cytosolic Ca^2+^ increases are impaired in granule neurons of *PS* cDKO mice, and that blockade of mitochondrial Ca^2+^ release mimics and occludes the Ca^2+^ homeostasis deficits in *PS* cDKO granule neurons. Taken together, our study demonstrates the importance of Presenilins in the regulation of synaptic plasticity and mitochondrial Ca^2+^ homeostasis at hippocampal MF synapses.

## Methods

### Mice

The generation and extensive characterization of postnatal forebrain-restricted *PS* conditional double knockout (*PS* cDKO) mice were previously reported [1, 2, 19, 20, 23]. Briefly, Northern, in situ hybridization and Western analyses were carried out to confirm the normal PS1 expression in *fPS1/fPS1* mice and the inactivation of PS1 in the cerebral cortex of *PS* cDKO (*fPS1/fPS1; PS2* ^*−/−*^ *; αCaMKII-Cre*) mice beginning at postnatal day ~18 and complete at ~4 weeks of age [1, 2, 19, 20]. *PS* cDKO (*fPS1/fPS1; PS2* ^*−/−*^ *; αCaMKII-Cre*) and control (*fPS1/fPS1*) mice were in the B6/129 hybrid background. All electrophysiological, Ca^2+^ imaging, quantitative EM and behavioral analyses were performed in a genotype blind manner using mice at the age of 2 months.

### Preparation of brain slices

Hippocampal slices were prepared from both male and female *PS* cDKO and control mice at 2 months of age. Mice were decapitated after being anesthetized with ketamine (100 mg/kg) + xylazine (10 mg/kg) + acepromazine (3 mg/kg), and the whole brains rapidly removed and placed in ice-cold (4 °C) oxygenated (95% O_2_/5% CO_2_) high sucrose and magnesium solution containing (in mM) the following: 200 Sucrose, 25 NaHCO_3_, 10 Glucose, 3 KCl, 1.25 NaH_2_PO_4_, 1.2 Na-pyruvate and 0.4 Na-ascorbate, 7 MgCl_2_, and 0.5 CaCl_2_. Horizontal hippocampal slices (400 μm thick) were prepared using a vibratome (VT1200S, Leica, Germany), and transferred to an incubation chamber having oxygenated artificial cerebrospinal fluid (ACSF) containing (in mM) the following: 125 NaCl, 3 KCl, 1.25 NaH_2_PO_4_, 1 MgCl_2_, 2 CaCl_2_, 25 NaHCO_3_, 10 Glucose, 1.2 Na-pyruvate and 0.4 Na-ascorbate, adjusted to 310 ± 5 mOsm (pH 7.4). The slices were allowed to recover at 34 °C for 30 min and then placed in a recording chamber constantly perfused with heated ACSF (30 ± 1 °C) and gassed continuously with 95% O_2_ and 5% CO_2_. The flow rates of bathing solution and the volume of the recording chamber for slices were 2.2 ml/min and 1.2 ml, respectively. Hippocampal slices were visualized using an upright microscope equipped with differential interference contrast (DIC) optics (BX51WI, Olympus, Japan). The DIC optics was used for visualization of neurons in the course of whole-cell recordings. All experiment procedures were conducted in accordance with guidelines of the ​Brigham and Women's Hospital Institutional Animal Care and Use Committee and National Institutes of Health.

### Electrophysiological analysis

For extracellular field recordings, stimulation pulses were delivered with a stimulus isolation unit (World Precision Instruments, A365) using a unipolar metal microelectrode. Stimulus electrodes were positioned ~600 μm from the recording electrode in the hilus adjacent to the DG granule cell layer (mossy fibers). Field excitatory postsynaptic potentials (fEPSPs) were recorded in current-clamp mode with ACSF-filled patch pipettes (1.5–2 MΩ). All fEPSPs were recorded with a stimulation strength that yielded 30% of the maximal response. To ensure that MF responses were not contaminated by associational/commissural inputs, the metabotropic glutamate receptor agonist (2S,1′R,2′R,3′R)-2-(2,3-dicarboxycyclopropyl) glycine (DCG IV; 2 μM) was applied at the end of recordings to block MF responses selectively. Data were included only if responses were reduced by more than 80%. All recordings were performed with the GABA_A_ receptor antagonist bicuculline methiodide (10 μM) and NMDA receptor antagonist APV (50 μM) added to the ACSF. Data were collected with a MultiClamp 700B amplifier (Molecular Devices) and digitized at 10 kHz using the A/D converter DIGIDATA 1322A (Molecular Devices). Data were acquired and analyzed using a custom program written with Igor Pro software (Version 6.3; Wave-Metrics) and Clampfit (Version 10.3; Molecular device).

For input/output measurements, 10 traces were averaged for each stimulation intensity, and the amplitude of the fiber volley (FV) was measured relative to the slope of the fEPSP. The stimulation rate was 0.2 Hz. The average linear fit slope was calculated as the slope of the linear input/output relationship for each slice. In LTP recordings, after baseline responses were collected every 15 s for 15 min, LTP was induced by five episodes of theta burst stimulation (TBS) delivered at 0.1 Hz. Each episode contained ten stimulus trains (5 pulses at 100 Hz) delivered at 5 Hz. To generate summary graphs (mean ± SEM), individual experiments were normalized to the baseline, and four consecutive responses were averaged to generate 1 min bins. These were then averaged together to generate the final summary graphs. Paired-pulse facilitation (PPF) was measured as the ratio of the second fEPSP slope relative to the first fEPSP slope, evoked by two identical presynaptic stimuli. Synaptic facilitation was measured as the percentage of the fEPSP slope versus the first fEPSP slope at a given stimulus train in individual slices.

For whole-cell patch clamp experiments, recording pipettes (3–5 MΩ) were filled with a solution containing (in mM) the following: 120 K-gluconate, 10 KCl, 20 HEPES, 4 MgATP, 0.3 NaGTP, 10 phosphocreatine, and 0.1 EGTA with the pH adjusted to 7.30 with KOH (295–300 mOsm). Excitatory postsynaptic currents (EPSCs) at MF synapses were recorded from CA3-pyramidal cells (CA3-PCs) in voltage-clamp mode at a holding potential of −60 mV. The series resistance (Rs) after establishing whole-cell configuration was between 15 and 20 MΩ. Synaptic responses were evoked by extracellular stimulation via a stimulator (Stimulus Isolator A365; WPI) connected to a patch electrode filled with ACSF solution, and placed in stratum lucidum of CA3 field. The stimulus intensity was adjusted such that the baseline EPSC amplitude was in the range between 100 pA and 300 pA. After 10–15 min of stabilization from the break-in, EPSCs were evoked with 0.2 Hz stimulation and recorded for 3–5 min, and followed by high frequency stimulation (HFS: 16 pulses at 100 Hz, delivered 4 times at 0.33 Hz) to induce post-tetanic potentiation (PTP). The PTP was recorded in the presence or absence of CGP37157 (20 μM), inhibitors of mitochondrial Na^+^/Ca^2+^ exchanger (NCX), and CGP37157 treatment began 3 min before second HFS and lasted during the PTP recording. The magnitude of PTP was quantified as the average of the first three post-tetanic EPSC amplitudes normalized to the mean baseline amplitudes. EPSC recordings with >20% series resistance change were excluded from data analysis. At the end of each experiment, we examined the effect of DCG IV (2 μM) to confirm that we had studied MF synapses.

### Quantitative EM

For quantitative EM analysis of mitochondria at MF synapses, four *PS* cDKO and four control mice at the age of 2 months were used. Animals were anaesthetized with sodium pentobarbital and perfused transcardially with physiological saline followed by fixative solution containing 1% glutaraldehyde and 4% paraformaldehyde in 0.1 M phosphate buffer (pH 7.4). Fixed brains were isolated and stored in fixative solution at 4 °C overnight, washed in phosphate buffer, and sectioned coronally on a vibratome (Leica VT 10005) at a thickness of 200 μm. Dorsal hippocampi were carefully excised and post-fixed in 1% OsO_4_ for 30 min. After rinsing in distilled water and dehydration in an ascending series of ethanol (block-staining with 0.5% uranyl acetate in 70% ethanol) followed by propylene oxide, the hippocampi were embedded in Epon (Fluka) and hardened at 65 °C for two days. Thin sections from *stratum lucidum* of area CA3 were cut on an ultratome (Leica Ultracut) and mounted on formvar-coated 50-mesh copper grids. Sections were post-stained with lead citrate and subjected to electron microscopy (6200× magnification). The number and area of mitochondria and the area of the presynaptic bouton profiles were quantified. To avoid multiple measurements of the same bouton, randomized sections 5 μm apart from each other were analyzed and at least 10 micrographs per mouse in the cohorts of four animals per condition were used.

### Calcium imaging

Cytosolic Ca^2+^ ([Ca^2+^]_i_) were measured in somata of hippocampal DG granule cells (GCs) using 100 μM Fura-2 pentapotassium salt introduced by a patch pipette. Ca^2+^ transients were evoked by 10 repetitive depolarizing pulses (2 ms duration from −80 to 0 mV) at various frequencies (1, 5, 10, and 20 Hz) under voltage-clamp conditions. For PTP induction, Ca^2+^ transients were elicited by 16 repetitive depolarizing pulses at 100 Hz, delivered 4 times at 0.33 Hz. Fluorescence imaging was performed with a 20× water-immersion objective (NA 0.5, UMPlanFln, Olympus), an air-cooling digital monochrome interline CCD camera (ORCA R2, Hamamatsu) and a monochromator (Polychrome V, FEI), which were controlled by a computer and Control Unit for real time TILL Imaging (FEI), running a Live Acqusition Imaging software. Images were taken at 20 Hz with double wavelength excitation at 340 and 380 nm. The ratio *r* = *F*_340_/*F*_380_ was converted to [Ca^2+^] values. Calibration parameters were determined using the in vitro calibration method (Invitrogen calibration kit). *R*_min_ and *R*_max_ values were calculated as 0.200 and 2.207, respectively.

### Behavioral analysis

Mice were housed in a standard 12 h light–dark cycle. Six *PS* cDKO and six control male mice at 2 months of age were used in the Morris water maze behavioral test. Mice were handled daily for 5 days before training or testing. The Morris water maze is a circular pool 160 cm in diameter. During water maze tasks, mice were trained for 13 days with four trials daily, and the probe tests were administered at days 7 and 13. Mice were released from all four quadrants in a pseudorandom manner during the four training trials. Four visible board cues were hung on the walls during training and probe tests. During the hidden platform training, the platform (10 cm in diameter) was kept submerged under water and maintained in the same position. Each mouse was given four trials daily with a maximum duration of 90 s separated by a minimum of 15 min. If mice did not find the hidden platform, they were guided to the platform and allowed to remain on it for 15 s. The swimming of the mice was monitored using an automated tracking system (HVS Image). After 6 days and 12 days training, two probe tests were performed in the morning of day 7 and day 13 before new training trials. During the probe test at day 7 and day 13, the hidden platform was removed and a 90 s probe test was performed. The mice were released from the opposite position of the platform and searching for the location of the hidden platform according to four visible board cues on the walls. After the 90 s probe test, mice were taken out of the pool and the platform was re-installed in the same position followed by four new training trails.

### Statistical analysis

Statistical analysis was performed using two-tailed Student’s *t*-test or two-way repeated-measures ANOVA with Bonferroni correction to test for significance for all comparisons of the electrophysiological, quantitative EM analysis, Ca^2+^ imaging and behavioral results. All data are presented as mean ± SEM.

## Results

### Impaired long-term potentiation at hippocampal MF synapses in *PS* cDKO mice

Despite the vulnerability of the hippocampal MF pathway in AD [17, 18], it was unknown whether Presenilins are involved in the regulation of synaptic function at MF synapses. We therefore performed electrophysiological recordings using acute hippocampal slices of forebrain-restricted *PS* cDKO mice at 2 months of age to investigate whether PS is required for synaptic transmission and plasticity at the MF pathway. We previously reported that Presenilins are inactivated in excitatory neurons of the hippocampus in these *PS* cDKO mice, beginning at postnatal day 18 and complete at ~1 month of age, and that there is no significant reduction of neurons and synapses in *PS* cDKO mice at 2 months of age [1–3, 19, 20].

**Fig. 1:**
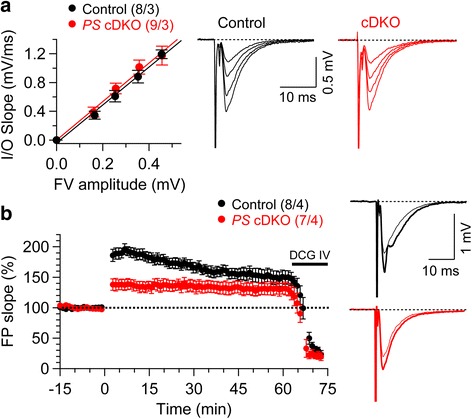
Impaired long-term potentiation at hippocampal MF synapses in *PS* cDKO mice. **a** Normal synaptic transmission in *PS* cDKO mice at 2 months of age. The synaptic input–output relationship was obtained by plotting the fiber volley (FV) amplitude against the initial slope of the evoked fEPSP. Each point represents data averaged across all slices for a narrow bin of FV amplitude. Right panel shows representative traces of fEPSPs evoked by various stimulation intensities. **b** Impaired LTP induced by 5 TBS in *PS* cDKO mice. Superimposed traces are averages of four consecutive responses 1 min before (*thin line*) and 50 min after (*thick line*) TBS induction. DCG IV (2 μM) was applied at the end of all experiments to confirm the recording of MF synapses. All data represent means ± SEM. The number of hippocampal slices (*left*) and mice (*right*) used in each experiment is indicated in *parentheses*

We first evaluated basal synaptic transmission by quantifying the initial slope of evoked field excitatory postsynaptic potential (fEPSP) and the amplitude of the fiber volley (FV), which is a measure of the number of recruited axons. Input/output (I/O) curves obtained by plotting the amplitude of FV versus the fEPSP slope in the presence of blockers of NMDA (50 μM APV) and GABA_A_ receptors (10 μM bicuculline) are similar between *PS* cDKO and control mice, indicating that basal synaptic transmission is normal in the absence of Presenilins (Fig. 1a). We further examined the effects of Presenilins inactivation on LTP at MF synapses. LTP induced by five trains of theta burst stimulation (TBS) is significantly impaired at MF synapses in *PS* cDKO mice (Fig. 1b). The magnitude of LTP measured during the last 10 min post-induction (50–60 min) is significantly lower in *PS* cDKO mice (130.9 ± 2.8%), relative to the control (150.5 ± 3.3%; unpaired *t*-test, *p* < 0.001). Furthermore, the magnitude of LTP measured during the first 10 min of recording is much lower in *PS* cDKO mice (136.9 ± 8.7%) relative to controls (189.6 ± 7.9%; unpaired *t*-test, *p* < 0.001). To determine whether the responses measured at the MF pathway might be contaminated with associational/commissural inputs, we continued the recording in the presence of the metabotropic glutamate receptor 2 (mGluR2) agonist DCG IV (2 μM), which suppresses transmission at MF synapses [24]. Indeed, LTP at MF synapses was blocked in the presence of DCG IV, confirming that the LTP measured was specific for MF synapses. Together, these results demonstrate that similar to SC synapses, Presenilins are essential for LTP induction at hippocampal MF synapses.

### Impaired short-term plasticity at hippocampal MF synapses in *PS* cDKO mice

**Fig. 2:**
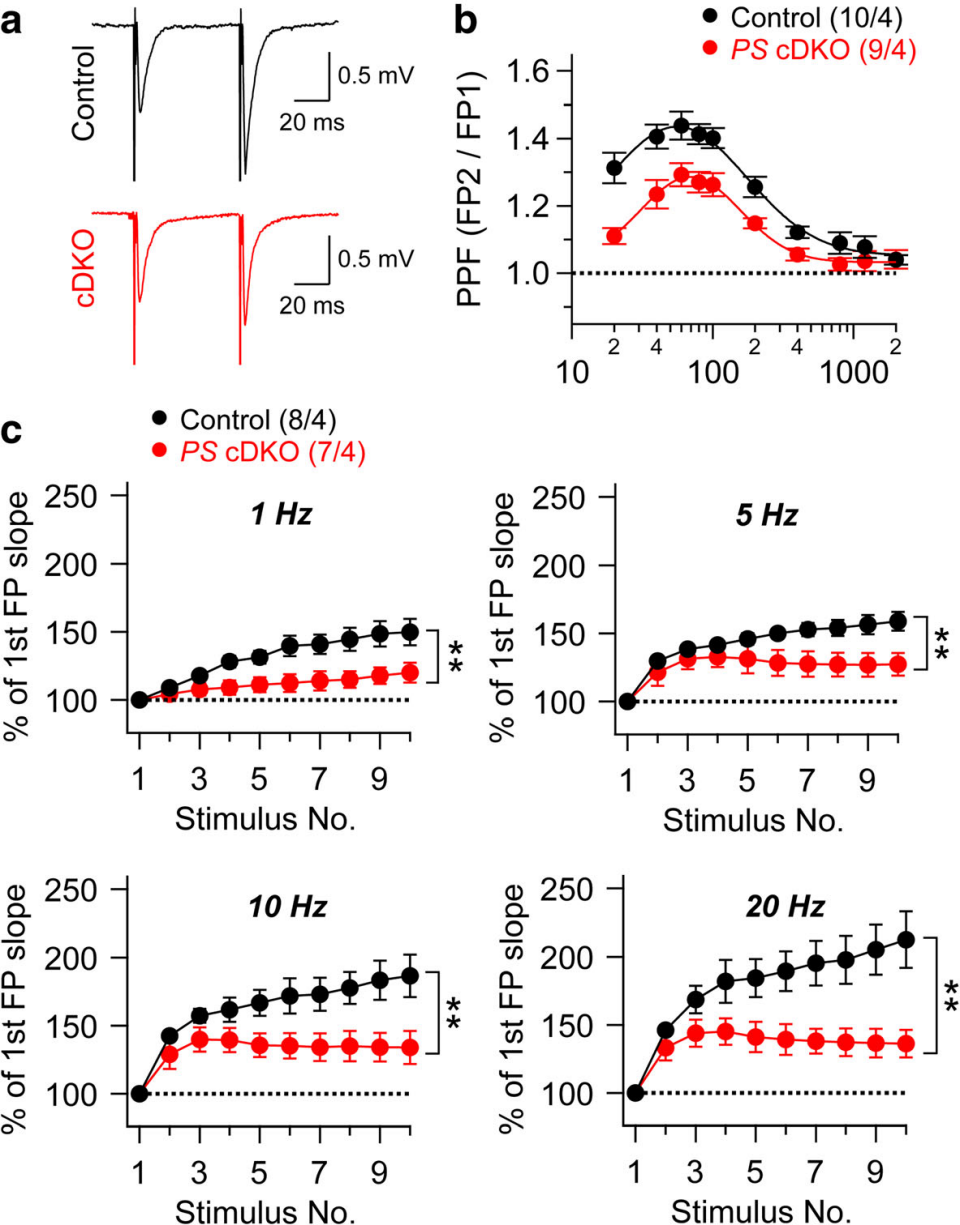
Impaired short-term plasticity at hippocampal MF synapses in *PS* cDKO mice. **a** Representative traces of fEPSPs evoked by two consecutive stimuli with a 60 ms inter-stimulus interval. **b** Average PPF plotted as a function of the inter-stimulus intervals (20-2000 ms) shows reduced PPF in *PS* cDKO mice (F_1, 17_ = 14.71; *p* = 0.0013; two-way ANOVA). **c** Synaptic facilitation elicited by stimulus trains is impaired in a frequency-dependent manner in *PS* cDKO mice (1 Hz: F_1, 13_ = 11.64, *p* = 0.005; 5 Hz: F_1, 13_ = 13.33, *p* = 0.003; 10 Hz: F_1, 15_ = 9.67, *p* = 0.008; 20 Hz: F_1, 15_ = 9.71, *p* = 0.008; two-way ANOVA). fEPSP slopes shown are normalized to the slope of the first fEPSP of the stimulus train. All data represent means ± SEM (** *p* < 0.01; two-way ANOVA). The number of hippocampal slices (*left*) and mice (*right*) used in each experiment is indicated in *parentheses*

Paired-pulse facilitation (PPF) and frequency facilitation are two forms of presynaptic short-term plasticity, which are induced by two closely spaced stimuli or short trains of higher frequency stimulation, respectively. To examine whether loss of PS function affects PPF and frequency facilitation in the hippocampal MF pathway, we recorded fEPSPs at MF synapses onto CA3 pyramidal neurons in *PS* cDKO mice. We found that PPF is significantly reduced in *PS* cDKO mice relative to the control, indicating impairment of short-term plasticity at MF synapses of *PS* cDKO mice (F_1, 17_ = 14.71; *p* = 0.0013; two-way ANOVA; Fig. 2a-b). Moreover, frequency facilitation induced by 10 stimuli applied at frequencies ranging from 1 to 20 Hz is also significantly reduced at MF synapses of *PS* cDKO mice (1 Hz: F_1, 13_ = 11.64, *p* = 0.005; 5 Hz: F_1, 13_ = 13.33, *p* = 0.003; 10 Hz: F_1, 15_ = 9.67, *p* = 0.008; 20 Hz: F_1, 15_ = 9.71, *p* = 0.008; two-way ANOVA; Fig. 2c), providing further evidence for pre-synaptic short-term plasticity impairment in these mice. Thus, Presenilins are required for normal presynaptic short-term plasticity at hippocampal MF synapses.

### Impaired post-tetanic potentiation at hippocampal MF synapses in *PS* cDKO mice

**Fig. 3:**
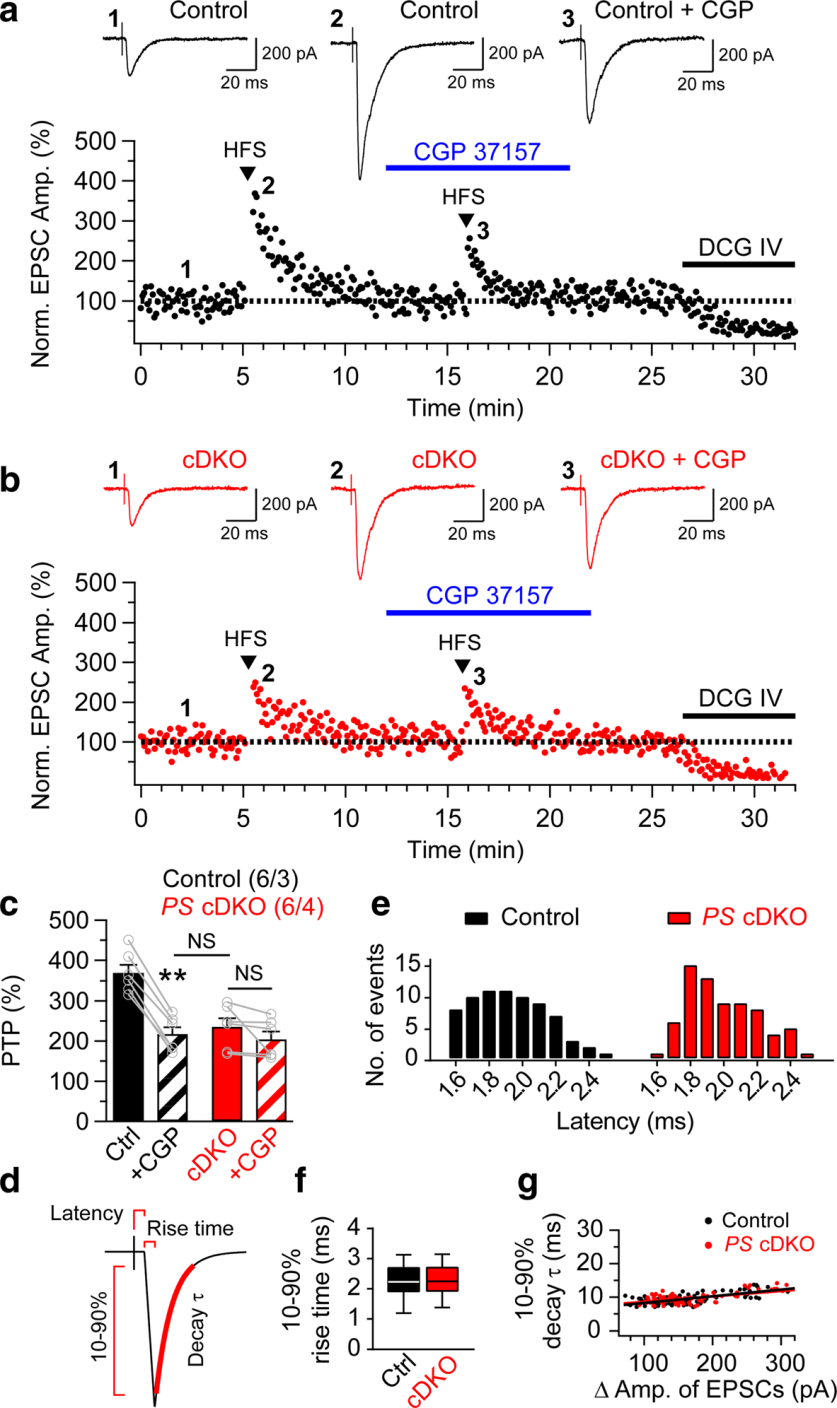
Impaired PTP at hippocampal MF synapses in *PS* cDKO mice. **a** & **b** Representative data showing the time course of PTP and the effect of CGP37157 (20 μM) on PTP of EPSCs obtained by whole-cell patch recording at the hippocampal MF synapses in control and *PS* cDKO mice. DCG IV (2 μM) was applied at the end of each experiment to confirm that MF synapses were recorded. The insets represent EPSC traces recorded the baseline (1) and immediately after HFS induction (2 & 3). Scale bar: 20 ms, 200 pA. **c** Summary bar graphs of the mean magnitude of PTP in the course of whole-cell patch recording in control and *PS* cDKO mice. PTP is impaired at MF synapses in *PS* cDKO mice. **d** Depicted EPSC trace for the estimation of latency, 10–90% rise time and decay time constant (τ). **e** Histogram of latency between stimulus onset and EPSC response was analyzed during 1 min baseline recordings of EPSC (*n* = 72 in 6 PTP recordings) in both control and *PS* cDKO mice. Latencies ranged from 1.6 to 2.5 ms with means of 1.93 ± 0.03 and 2.01 ± 0.03 ms in the control and *PS* cDKO mice, respectively. **f** The 10–90% rise time for control EPSCs (2.27 ± 0.06 ms range from 1.19 to 3.13 ms, *n* = 72) and *PS* cDKO EPSCs (2.29 ± 0.05 msec range from 1.38 to 3.14 ms, *n* = 72) is shown. **g** Relationship of 10–90% decay time constant (τ) and Δ amplitude of EPSCs. The distribution was well fitted with linear function (*Black dots* for control: y = 0.019 x + 6.288, *n* = 72; *red dots* for *PS* cDKO: y = 0.016 x + 7.005, *n* = 72). All data represent means ± SEM (** *p* < 0.01, NS: not significant; Student’s *t*-test). The values in *parentheses* indicate the number of hippocampal neurons (*left*) and the number of mice (*right*) used in each experiment

Other forms of short-term synaptic plasticity also include post-tetanic potentiation, which lasts seconds to several minutes and is longer-lasting than the time scale of hundreds of milliseconds for frequency facilitation. PTP is known to require mitochondrial Ca^2+^ for its induction [21, 22, 25, 26]. We examined PTP in the presence of APV (50 μM) and bicuculline (10 μM) at hippocampal MF synapses of *PS* cDKO and control mice using whole-cell patch clamp recording in CA3 pyramidal neurons. After baseline EPSC amplitudes were recorded for 5 min at 0.2 Hz, PTP was induced by high frequency stimulation (HFS: 16 pulses at 100 Hz, delivered 4 times at 0.33 Hz), and time-dependent changes in the EPSC amplitudes were measured. Figure 3a and b show representative time courses of the changes in normalized EPSC amplitudes relative to the baseline responses recorded from CA3 pyramidal neurons of control and *PS* cDKO mice. We found that PTP recorded in the MF pathway is reduced in CA3 pyramidal neurons of *PS* cDKO mice, relative to controls (Fig. 3c). Application of CGP37157 (20 μM), inhibitor of mitochondrial Ca^2+^ release via the Na^+^/Ca^2+^ exchanger (NCX), resulted in significant reduction of PTP at MF synapses of control mice, whereas CGP37157 treatment had little effect on PTP at MF synapses in *PS* cDKO mice (Fig. 3a-c). DCG IV (2 μM) was applied towards the end of the recording to confirm that MF synapses were recorded. These results show that PTP induction is impaired in the absence of Presenilins at MF synapses, and that blockade of mitochondrial Ca^2+^ release by CGP37157 in control mice mimics and occludes the PTP reduction in *PS* cDKO mice.

To confirm further whether synaptic inputs indeed arise from monosynaptic MF innervation, we next analyzed EPSC characteristics as previously reported [27, 28]. We found that synaptic latencies to the EPSC onset showed unimodal distribution and their range was 1.6–2.5 ms, with averages of 1.93 ± 0.03 and 2.01 ± 0.03 ms in control and *PS* cDKO mice, respectively (Fig. 3e). In addition, we measured kinetic parameters of EPSCs (10–90% rise time and decay time constant of EPSCs) to ensure that analysis was restricted to MF inputs. The mean 10–90% rise times are 2.27 ± 0.06 and 2.29 ± 0.05 ms in the range of 1.19–3.13 and 1.38–3.14 ms in control and *PS* cDKO mice, respectively (Fig. 3f). Furthermore, we found that the 10–90% decay time constant (τ) of EPSCs does not correlate with the EPSC amplitude (Fig. 3g). Taken together, these findings indicate that EPSC recordings in the MF pathway reflected monosynaptic responses.

### Quantitative EM analysis of presynaptic MF boutons

**Fig. 4:**
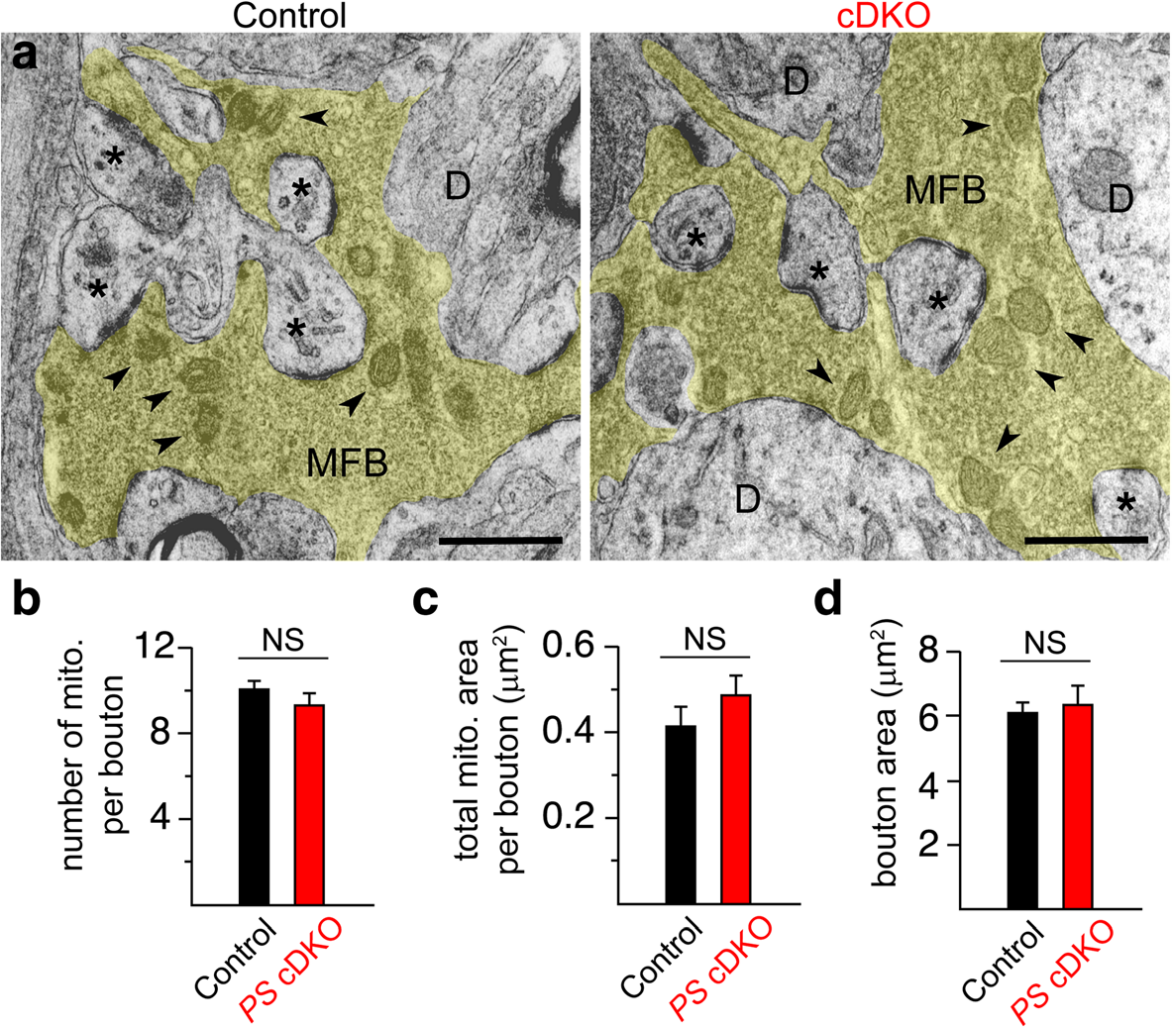
Normal mitochondrial content at presynaptic MF boutons of *PS* cDKO mice. **a** Transmission electron micrographs of hippocampal *stratum lucidum* in area CA3 show large presynaptic mossy fiber boutons (MFB: *highlighted in yellow*) establishing synaptic contacts with large complex spines (*asterisks*) of CA3 pyramidal neuron dendrites (D). The *arrowheads* indicate mitochondria in the MFB. Scale bars: 1 μm. The randomized samples were obtained from at least 10 micrographs from each of the 4 mice per genotype. **b**-**d** Quantification of the number of mitochondria per bouton (control: 10.02 ± 0.33, *PS* cDKO: 9.51 ± 0.33, *n* = 59 boutons), total mitochondrial area per bouton (control: 0.42 ± 0.04 μm^2^, *n* = 44; *PS* cDKO: 0.49 ± 0.04 μm^2^, *n* = 42), and bouton area (control: 6.04 ± 0.45 μm^2^, *n* = 59; *PS* cDKO: 6.41 ± 0.58 μm^2^, *n* = 42). The data are presented as means ± SEM (NS: not significant, Student’s *t*-test)

Our findings showing that blockade of mitochondrial Ca^2+^ release in control mice mimics and occludes PTP impairment at MF synapses in *PS* cDKO mice suggest deficits in mitochondrial Ca^2+^ homeostasis in the absence of Presenilins. To determine whether mitochondrial content is altered at presynaptic MF boutons of *PS* cDKO mice, we performed quantitative EM analysis of *PS* cDKO and control mice at 2 months of age. MF synapses are known to exhibit unique ultrastructural characteristics compared to other hippocampal synapses, including large presynaptic boutons [29–31]. As shown in Fig. 4a, large numbers of mitochondria are present in presynaptic boutons of *PS* cDKO and control MF synapses. Quantitative analysis of mitochondrial profiles revealed similar numbers of mitochondria per bouton between control and *PS* cDKO mice (control: 10.02 ± 0.33, *PS* cDKO: 9.51 ± 0.33, *n* = 59 boutons, *p* > 0.05; Fig. 4b). Total area of mitochondria per bouton is also similar between control and *PS* cDKO MF synapses (control: 0.42 ± 0.04 μm^2^, *n* = 44 boutons; *PS* cDKO: 0.49 ± 0.04 μm^2^, *n* = 42 boutons, *p* > 0.05; Fig. 4c). Furthermore, the area of the presynaptic MF bouton is normal in *PS* cDKO mice (control: 6.04 ± 0.45 μm^2^, *n* = 59 boutons; *PS* cDKO: 6.41 ± 0.58 μm^2^, *n* = 42 boutons, *p* > 0.05; Fig. 4d). Thus, mitochondrial content is not changed at hippocampal MF presynaptic terminals in the absence of Presenilins.

### Impaired mitochondrial Ca^2+^homeostasis in *PS* cDKO granule neurons

**Fig. 5:**
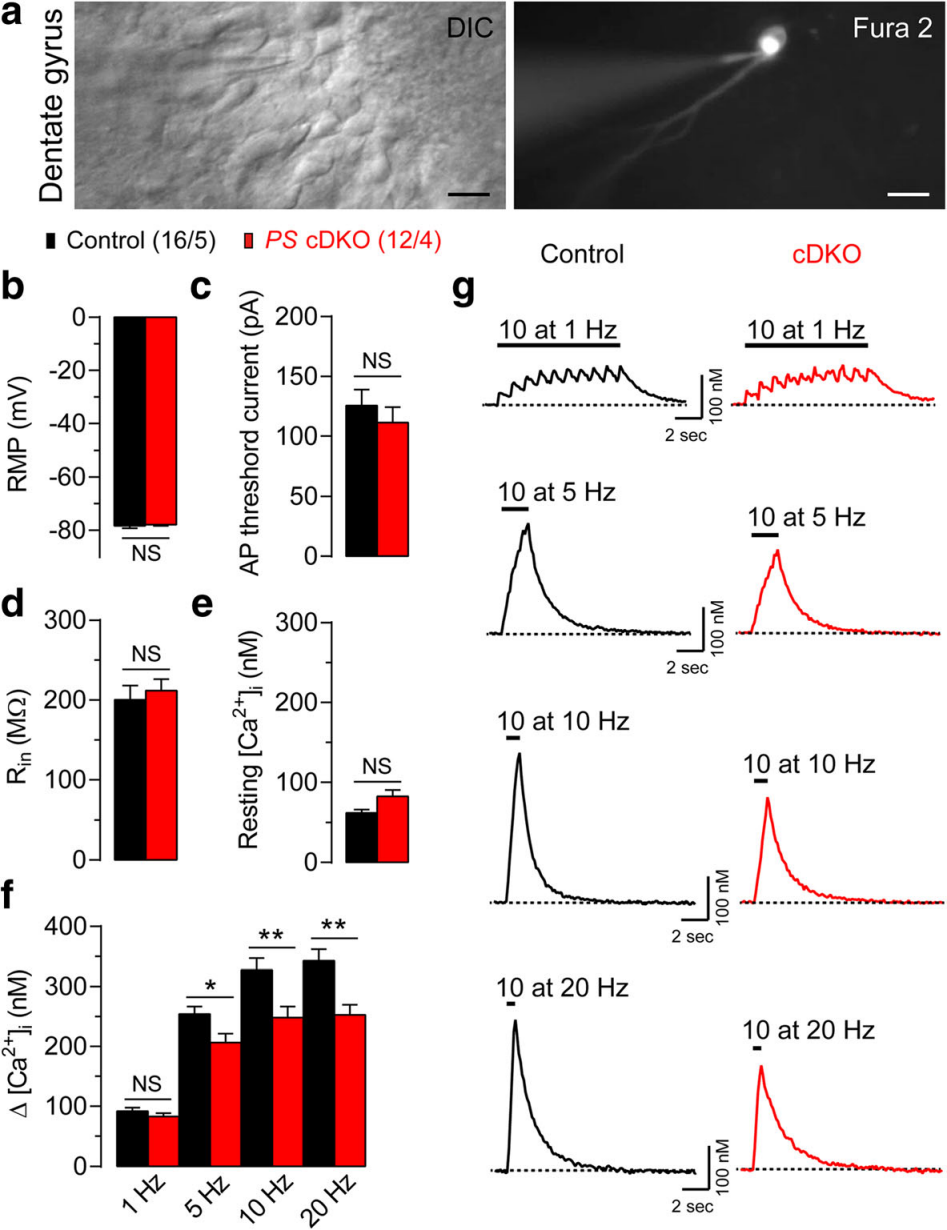
Reduced cytosolic Ca^2+^ increases induced by repetitive stimulation in hippocampal DG granule neurons of *PS* cDKO mice. **a** (*Left*) DIC image of DG granule neurons in an acute hippocampal slice in the course of whole-cell recording. (*Right*) Fluorescence image obtained from a soma of DG granule neurons loaded with 100 μM Fura 2 via a whole-cell patch pipette. Scale bars: 20 μm. **b**-**d** Electrophysiological characteristics of mature DG granule neurons: mean values for resting membrane potential (Control: −78.25 ± 0.94 mV; *PS* cDKO: −77.83 ± 0.41 mV), threshold currents for triggering of action potential (Control: 125.7 ± 12.3 pA; *PS* cDKO: 111.4 ± 11.9 pA) and input resistance (Control: 200.4 ± 16.5 MΩ; *PS* cDKO: 211.8 ± 13.4 MΩ). **e** Resting Ca^2+^ level (Control: 62.1 ± 4.1 nM; *PS* cDKO: 82.9 ± 7.4 nM) is not significantly different between control and *PS* cDKO mice. **f** The amplitude of ∆[Ca^2+^]_i_ elicited by 10 repetitive stimulation (depolarizing pulses of 2 ms duration; from −80 to 0 mV) at 5, 10, 20 Hz is reduced in *PS* cDKO granule neurons. **g** Representative Ca^2+^ transients evoked by 10 repetitive stimulation at various frequencies (1, 5, 10, and 20 Hz). Scale bars: 2 s, 100 nM. All data represent means ± SEM (* *p* < 0.05, ** *p* < 0.01, NS: not significant; Student’s *t*-test). The values in *parentheses* indicate the number of neurons (*left*) and the number of mice (*right*) used in each experiment

To evaluate Ca^2+^ homeostasis directly in DG granule neurons, which are the presynaptic neuron of the MF synapse, we used acute hippocampal slices to measure cytosolic Ca^2+^ concentration ([Ca^2+^]_i_) in DG granule neurons upon repeated stimulations at the same frequencies that were used to induce synaptic facilitation (Fig. 2c). We imaged the fluorescence of Fura-2 in the soma, which was introduced at a concentration of 100 μM via a whole-cell patch pipette (Fig. 5a). Before examining Ca^2+^ responses, we identified mature DG granule neurons in the hippocampus according to their electrophysiological criteria: low resting membrane potential (RMP) and input resistance (R_i_), and high threshold current for action potential compared to young DG granule neurons [32, 33]. The mean values for RMP, R_i_, and threshold currents for triggering action potential are similar between control and *PS* cDKO granule neurons (Fig. 5b-d; *p* > 0.05). Ca^2+^ transients were evoked by 10 repetitive stimulation (depolarizing pulses of 2 ms duration; from −80 to 0 mV) at varying frequencies (1, 5, 10, and 20 Hz) under voltage-clamp conditions. The resting [Ca^2+^]_i_ is not significantly different between control (62.1 ± 4.1 nM; *n* = 16 neurons) and *PS* cDKO (82.9 ± 7.4 nM; *n* = 12 neurons, unpaired *t*-test, *p* = 0.102) DG neurons (Fig. 5e). However, the magnitude of [Ca^2+^]_i_ changes (∆[Ca^2+^]_i_) elicited by 10 repetitive stimulation at 5, 10 and 20 Hz are significantly reduced in DG granule neurons of *PS* cDKO mice, compared to the control (*p < 0.05, **p < 0.01; Fig. 5f-g). These findings suggest that presynaptic Ca^2+^ deficits may contribute to or underlie the presynaptic facilitation impairment observed at MF synapses in *PS* cDKO mice (Fig. 2c).

**Fig. 6:**
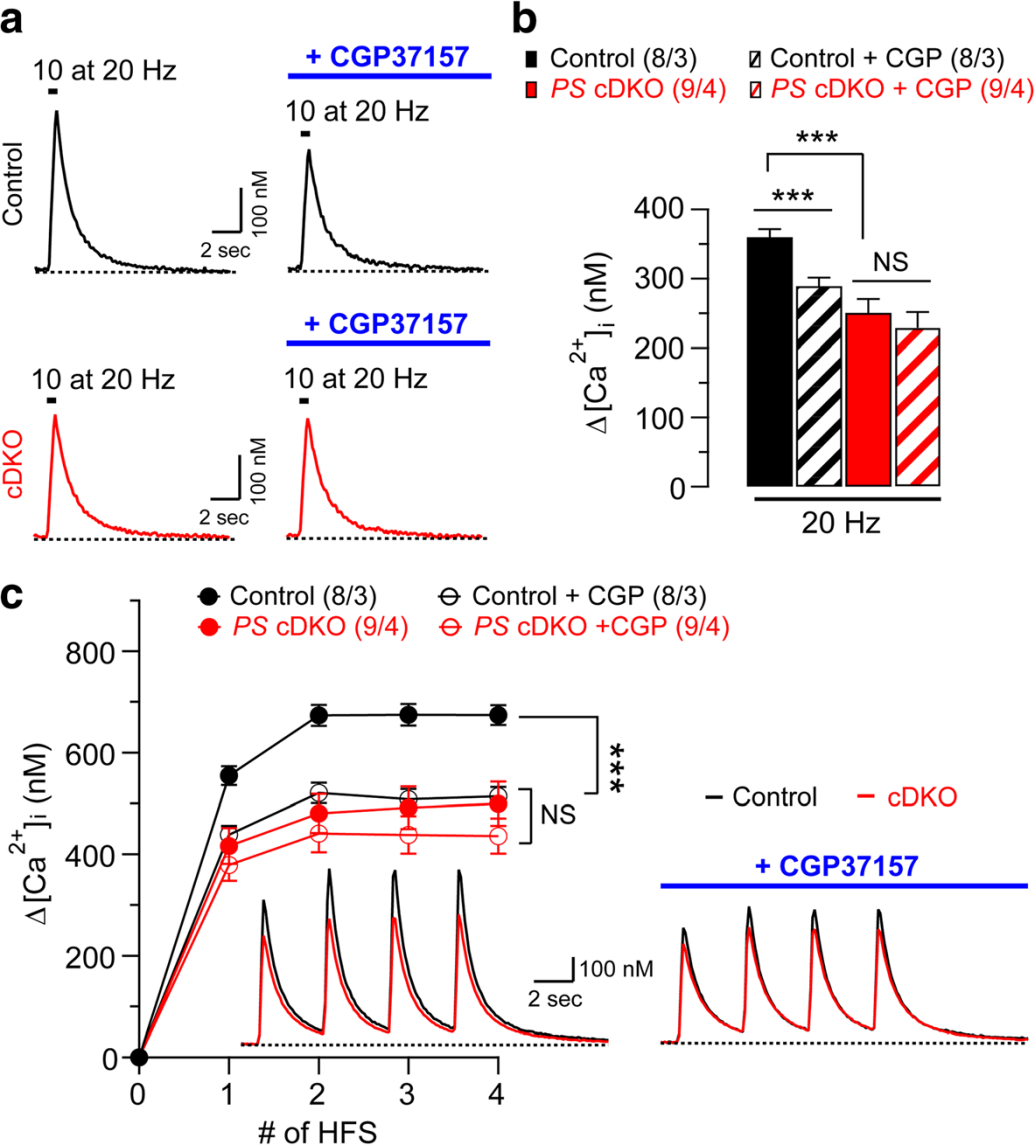
Inhibition of mitochondrial Ca^2+^ release mimics and occludes deficits of mitochondrial Ca^2+^ homeostasis in hippocampal DG granule neurons of *PS* cDKO mice. **a** Representative Ca^2+^ transients evoked by 10 repetitive stimulation (depolarizing pulses of 2 ms duration; from −80 to 0 mV) at 20 Hz recorded in the absence or presence of CGP37157. Scale bar: 2 s, 100 nM. **b** Summary bar graph of the amplitude of ∆[Ca^2+^]_i_ shows significant reduction in *PS* cDKO granule neurons (250.7 ± 19.7 nM, unpaired *t*-test, *p* < 0.001) and control granule neurons treated with CGP37157 (289.2 ± 12.1 nM, paired *t*-test, *p* < 0.001), relative to untreated control neurons (360.1 ± 11.4 nM). Bar graphs represent means ± SEM (*** *p* < 0.001; Student’s *t*-test, NS: not significant). **c** The amplitude of ∆[Ca^2+^]_i_ elicited by PTP inducing stimulation (16 pulses at 100 Hz, 4 times delivered at 0.33 Hz; from −80 to 0 mV) is significantly reduced in *PS* cDKO granule neurons and control granule neurons treated with CGP37157, relative to untreated control neurons (control vs *PS* cDKO: F_1, 15_ = 12.56, *p* = 0.0029, control vs control + CGP: F_1, 14_ = 28.46, *p* = 0.0001; two-way ANOVA). CGP37157 treatment does not significantly reduce ∆[Ca^2+^]_i_ in *PS* cDKO neurons (F_1, 16_ = 0.76, *p* = 0.40; two-way ANOVA). The insets are averaged Ca^2+^ transients. The values in *parentheses* indicate the number of neurons (*left*) and the number of mice (*right*) used in each experiment

To determine further whether the reduction of cytosolic Ca^2+^ increases in presynaptic neurons of MF synapses in *PS* cDKO mice is due to mitochondrial Ca^2+^ dysregulation, we examined the amplitude of [Ca^2+^]_i_ changes elicited by repetitive stimulation in the absence or presence of CGP37157 (20 μM) at DG granule neurons of *PS* cDKO and control mice. We found that the magnitude of ∆[Ca^2+^]_i_ induced by repetitive stimulation (10 depolarizing pulses of 2 ms duration; from −80 to 0 mV) at 20 Hz is significantly reduced in *PS* cDKO granule neurons, relative to the control (control: 360.1 ± 11.4 nM, *PS* cDKO: 250.7 ± 19.7 nM, unpaired *t*-test, *p* = 0.0003; Fig. 6a-b). In the presence of CGP37157, ∆[Ca^2+^]_i_ in DG granule neurons of control mice is significantly reduced (control: 360.1 ± 11.4 nM, control + CGP: 289.2 ± 12.1 nM, paired *t*-test, *p* = 0.0008; Fig. 6a-b). However, CGP37157 treatment did not cause further significant reduction of ∆[Ca^2+^]_i_ in DG granule neurons of *PS* cDKO mice (*PS* cDKO: 250.7 ± 19.7 nM, *PS* cDKO + CGP: 230.4 ± 22.2 nM, paired *t*-test, *p* = 0.482; Fig. 6a-b). Furthermore, ∆[Ca^2+^]_i_ induced by the PTP induction protocol (16 pulses at 100 Hz, delivered 4 times at 0.33 Hz) is also significantly reduced in *PS* cDKO granule neurons, relative to the control (F_1, 15_ = 12.56, *p* = 0.0029; two-way ANOVA; Fig. 6c), and inhibition of mitochondrial Ca^2+^ release by CGP37157 significantly reduces the amplitude of ∆[Ca^2+^]_i_ during PTP induction in control mice (F_1, 14_ = 28.46, *p* = 0.0001; two-way ANOVA), but does not significantly reduce ∆[Ca^2+^]_i_ in *PS* cDKO mice (F_1, 16_ = 0.76, *p* = 0.40; two-way ANOVA; Fig. 6c). These results show that mitochondrial Ca^2+^ homeostasis is disrupted in the absence of Presenilins, and suggest that mitochondrial Ca^2+^ deficits in presynaptic DG granule neurons underlie the short-term plasticity impairment observed at MF synapses in *PS* cDKO mice.

### Spatial memory impairments in *PS* cDKO mice

**Fig. 7:**
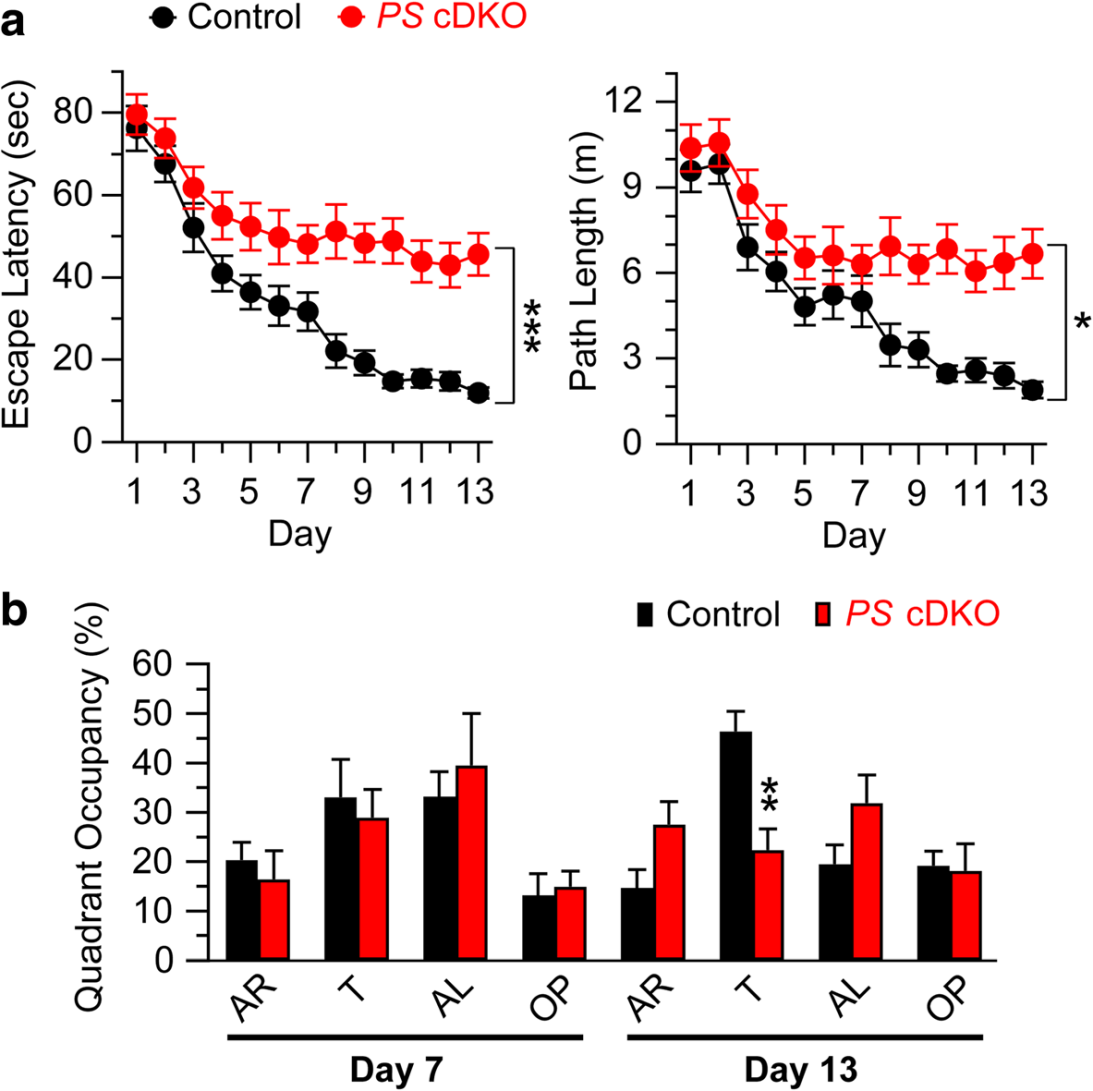
Impaired spatial learning and memory in *PS* cDKO mice at 2 months of age. **a** Escape latency of *PS* cDKO mice (*n* = 6) and controls (*n* = 6) gradually decreases during 13 days of training in the hidden platform water maze task, and the latency is significantly higher in *PS* cDKO mice (F_1, 10_ = 22.69, *p* < 0.001; two-way ANOVA). Path length is also gradually decreased for both *PS* cDKO and control mice during training, and the path length of *PS* cDKO mice is significantly longer, relative to control mice (F_1, 10_ = 9.62, *p* < 0.05; two-way ANOVA). **b** Two post-training probe trials were performed on days 7 and 13. Both *PS* cDKO and control mice show similar target quadrant occupancy on day 7, but *PS* cDKO mice show significantly reduced target quadrant occupancy on day 13 (*p* < 0.01; Student’s *t*-test). Post-hoc power analysis showed that 6 mice results in 99%, 99% and 98% power for latency, path length, and quadrant occupancies, respectively, at day 13. AR: adjacent right quadrant, T: target quadrant, AL: adjacent left quadrant, OP: opposite quadrant. All data are means ± SEM. The *asterisks* denote statistical significance (* *p* < 0.05, ** *p* < 0.01, *** *p* < 0.001)

We further assessed *PS* cDKO and control mice in the Morris water maze using a difficult training protocol (4 trials a day; 13 days). The performance of both control and *PS* cDKO mice improved significantly during the course of training (day 1 vs. day 13, *p* < 0.001; Fig. 7a). Two-way ANOVA analysis revealed that *PS* cDKO mice exhibit significantly longer latencies (F_1_, _10_ = 22.69, *p* < 0.001) and path lengths (F_1_, _10_ = 9.62, *p* < 0.05) relative to control mice (Fig. 7a), while the swimming speed was similar (F_1_, _10_ = 4.55, *p* > 0.05; data not shown). In the post-training probe trial on day 13, both control and *PS* cDKO mice searched preferentially in the target quadrant, but *PS* cDKO mice showed significantly reduced target quadrant occupancy (*p* < 0.01; Fig. 7b). These results demonstrate that *PS* cDKO mice exhibit profound spatial learning and memory impairment in the water maze.

## Discussion

The hippocampus is known to be particularly vulnerable in AD, and is composed of three major circuits [7–9, 17, 18]. Our previous studies of Presenilins and Nicastrin, another essential component of the γ-secretase complex, in synaptic function, however, focused exclusively on the hippocampal SC pathway [1, 2, 19, 34]. In the current study, we investigate the role of Presenilins at hippocampal MF synapses to determine whether Presenilins employ a universal or distinct mechanism to control synaptic function in the hippocampus. Our electrophysiological, quantitative EM and imaging analyses revealed the essential role of Presenilins in the regulation of synaptic plasticity and mitochondrial Ca^2+^ homeostasis at hippocampal MF synapses.

Similar to SC synapses, we found that presynaptic short-term plasticity, such as PPF and synaptic facilitation, and LTP are impaired at hippocampal MF synapses in the absence of Presenilins (Figs. 1 and 2), indicating a universal requirement of Presenilins for normal synaptic plasticity at the hippocampal SC and MF synapses. These findings are consistent with the spatial learning and memory deficits exhibited by these *PS* cDKO mice at 2 months of age in the hippocampal memory-dependent Morris water maze task using either an intensive training protocol (6 trials per day) in a prior study [1] or a less intensive and more difficult training protocol (4 trials per day) in the current study, which revealed more dramatic learning and memory deficits (Fig. 7). Interestingly, *PS1* cKO mice also exhibited mild but significant learning and memory deficits using a similarly difficult training protocol, which was designed to uncover more readily spatial learning and memory impairment [20]. Thus, the severity of learning and memory deficits observed in the water maze is Presenilin dose dependent with *PS* cDKO mice exhibit more severe phenotypes than *PS1* cKO mice. The synaptic plasticity impairments observed in the MF pathway of *PS* cDKO mice in the current study and previously reported in the SC pathway [1, 2, 19] likely contribute to the spatial learning and memory deficits identified in the current and prior studies (Fig. 7, [1]). For example, MF synaptic plasticity was reported to be important for the establishment of hippocampus-dependent associative learning and spatial memory [35–38]. Furthermore, spatial learning and memory analyzed in the water maze results from network interactions between hippocampal tri-synaptic circuits and the entorhinal cortex. At 2 months of age, postnatal forebrain-restricted *PS* cDKO mice exhibit normal numbers of cortical and hippocampal neurons as well as normal volume of the neocortex and hippocampus, indicating unaffected cortical development in these mice [1, 3], despite neurodevelopment phenotypes observed in *PS* germ-line mutant mice and neural progenitor cell lineage restricted conditional mutant mice [39–42]. Future studies will be needed to determine whether and how Presenilins control synaptic plasticity in the hippocampal perforant path (PP).

The impairment in the LTP induction phase is more dramatic at MF synapses of *PS* cDKO mice (Fig. 1) than what was previously reported at SC synapses [2, 43]. This is likely due to the fact that high-frequency stimulation at MF synapses induces multiple forms of synaptic strength enhancements, including PTP, in LTP induction phase [44, 45]. Furthermore, in contrast to the SC and the perforant path, MF synapses display a particular form of LTP that is mainly expressed presynaptically, and is independent of NMDA receptor activation [45–47]. The early induction phase of LTP at MF synapses is triggered by a tetanus-induced rise in presynaptic intracellular Ca^2+^, which results in activation of a Ca^2+^/calmodulin-activated adenylyl cyclase [47–50]. The mitochondrial Ca^2+^ deficit observed in presynaptic neurons of the MF pathway in the absence of Presenilins (Fig. 6) likely underlies the greater impairment of early phase LTP induction at MF synapses relative to SC synapses.

Interestingly, we found that another form of presynaptic short-term plasticity, PTP, is impaired at MF synapses in *PS* cDKO mice (Fig. 3). Due to their unique structural features, CA3 pyramidal neurons receive excitatory synapses from stellate cells of layer II of the entorhinal cortex onto their distal apical dendrite [51, 52], and from other CA3 axon collaterals onto the remainder of the apical and the entire basal dendrite [53]; thus, the MF-CA3 projection may be contaminated with polysynaptic responses. We therefore performed whole-cell patch recording to ensure that the EPSCs recorded at MF synapses were monosynaptic. The latter is supported by the observations that the EPSC’s rise times are uniform, and their latencies are relatively short, and their distribution is unimodal (Fig. 3). Furthermore, if our EPSC recording were contaminated with polysynaptic contributions, then increasing the intensity of presynaptic stimulation would expect to result in slower-decaying synaptic currents. However, we found that the EPSCs decay time constant (τ) did not correlate with the EPSC amplitude or stimulation intensity (Fig. 3). This is consistent with monosynaptic nature of the recorded EPSCs [27, 28], and suggests that our EPSC recording in the MF pathway reflected monosynaptic responses with no significant contamination by polysynaptic components.

PTP is known to be dependent on mitochondrial Ca^2+^ and is longer lasting than frequency facilitation due to the slower release of Ca^2+^ from mitochondria [21, 22, 25, 54]. Indeed, blockade of mitochondrial Ca^2+^ release by NCX inhibitor CGP37157 mimics and occludes the PTP impairment observed at MF synapses of *PS* cDKO mice (Fig. 3), indicating that the PTP deficits in *PS* cDKO mice are due to the mitochondrial Ca^2+^ defects. However, quantitative EM analysis revealed similar number and area of mitochondria at presynaptic boutons of control and *PS* cDKO MF synapses (Fig. 4). Ca^2+^ imaging analysis of acute hippocampal slices demonstrated that Presenilins are essential for normal mitochondrial Ca^2+^ homeostasis at MF synapses (Figs. 5 and 6). We measured the ∆[Ca^2+^]_i_ increments in the cell body of DG granule neurons instead of presynaptic axon terminals, and the cytosolic Ca^2+^ increases are reduced in DG granule neurons of *PS* cDKO mice when induced by tetanic stimulation between 5 and 20 Hz but unchanged at 1 Hz (Fig. 5). However, synaptic facilitation induced by repeated stimulation at 1, 5, 10 and 20 Hz is impaired at MF synapses of *PS* cDKO mice (Fig. 2). The difference between cytosolic Ca^2+^ increases and synaptic facilitation induced by repeated stimulation at 1 Hz is likely due to the fact that during stimulation [Ca^2+^]_i_ increments are much higher in presynaptic axon terminals and have faster kinetics, compared to cell bodies, because of their different Ca^2+^ clearance mechanisms and endogenous Ca^2+^ buffers [55–60].

Blockade of mitochondrial Ca^2+^ release mimics the impairment of cytosolic Ca^2+^ increases elicited by PTP induction stimuli (16 pulses at 100 Hz, delivered 4 times) in DG granule neurons of *PS* cDKO mice (Fig. 6). Furthermore, the amplitude of cytosolic Ca^2+^ increases elicited by high frequency stimulation (>20 Hz) is similarly reduced in DG granule neurons of *PS* cDKO slices and in DG granule neurons of control slices treated with CGP37157, whereas CGP37157 has little effect in *PS* cDKO DG neurons (Fig. 6). These results suggest that the mitochondrial Ca^2+^ deficits likely contribute to the presynaptic impairment observed at MF synapses of *PS* cDKO mice. How Presenilins control mitochondrial Ca^2+^ homeostasis is unknown. We previously reported that ryanodine receptor (RyR)-mediated Ca^2+^ release from the ER is impaired in the absence of Presenilins [2, 61]. Furthermore, RyR levels are reduced in the hippocampus of *PS* cDKO mice but IP_3_ receptors and SERCA are unchanged [61]. It remains to be determined whether Presenilin regulates mitochondrial homeostasis via its uniporter and/or antiporters or through its modulation of Ca^2+^ release from the ER, since communication between ER and mitochondrial membranes is thought to facilitate Ca^2+^ transfer [62–64]. Since mitochondrial Ca^2+^ dysregulation likely contributes to apoptotic neuronal death observed in *PS* cDKO mice during aging [3], future studies will aim at elucidation of the molecular mechanism by which Presenilins control mitochondrial Ca^2+^ homeostasis, which may be explored to prevent neurodegeneration caused by Presenilin dysfunction.

## Conclusions

Our prior studies addressing the normal synaptic function of Presenilins and the dysfunction of Presenilin mutations all focused on the hippocampal Schaffer collateral pathway. Little is known about the function and dysfunction of Presenilins in other hippocampal synapses, such as the mossy fiber pathway, which are also vulnerable in the pathogenesis of Alzheimer’s disease. In this study, we report that loss of Presenilin function leads to impairment in long-term potentiation and multiple forms of presynaptic short-term plasticity at hippocampal mossy fiber synapses. Interestingly, mitochondrial Ca^2+^ homeostasis is also disrupted at mossy fiber synapses in the absence of Presenilin, though mitochondrial content is unaffected, and the presynaptic Ca^2+^ dysregulation likely contributes to the presynaptic impairment observed at mossy fiber synapses in *PS* cDKO mice. Our current study demonstrates the importance of Presenilin in the regulation of synaptic plasticity and mitochondrial Ca^2+^ homeostasis in the hippocampal mossy fiber pathway.

## Abbreviations

AD: Alzheimer’s disease
DG: Dentate gyrus
EM: Electron microscopy
EPSC: Excitatory postsynaptic currents
fEPSP: Field excitatory postsynaptic potentials
FV: Fiber volley
HFS: High frequency stimulation
LTP: Long-term potentiation
MF: Mossy fiber
NCX: Na^+^/Ca^2+^ exchanger
PP: Perforant path
PPF: Paired-pulse facilitation
*PS* cDKO: *Presenilin* conditional double knockout
PS: Presenilin
PTP: Post-tetanic potentiation
R_i_: Input resistance
RMP: Resting membrane potential
RyR: Ryanodine receptor
SC: Schaffer collateral
SERCA: Sarcoendoplasmic reticulum Ca^2+^-ATPase
TBS: Theta burst stimulation
